# 5-Fluorouracil/L-Leucovorin Plus Oxaliplatin (FOLFOX) Regimen as Salvage Chemotherapy for Patients with Unresectable Pancreatic Cancer Receiving Gemcitabine and Nab-Paclitaxel and 5-Fluorouracil/L-Leucovorin Plus Nanoliposomal Irinotecan: Preliminary Results from Clinical Practice

**DOI:** 10.3390/curroncol29040216

**Published:** 2022-04-11

**Authors:** Takuo Yamai, Kenji Ikezawa, Yasuharu Kawamoto, Takeru Hirao, Sena Higashi, Kazuma Daiku, Shingo Maeda, Yutaro Abe, Makiko Urabe, Yugo Kai, Ryoji Takada, Tasuku Nakabori, Hiroyuki Uehara, Kazuyoshi Ohkawa

**Affiliations:** Department of Hepatobiliary and Pancreatic Oncology, Osaka International Cancer Institute, 3-1-69 Otemae, Chou-ku, Osaka 541-8567, Japan; takuo.yamai@oici.jp (T.Y.); yasuharu.kawamoto@oici.jp (Y.K.); takeru.hirao@oici.jp (T.H.); sena.higashi@oici.jp (S.H.); kazuma.daiku@oici.jp (K.D.); shingo.maeda@oici.jp (S.M.); abe-yu@mc.pref.osaka.jp (Y.A.); makiko.urabe@oici.jp (M.U.); yugo.kai@oici.jp (Y.K.); ryoji.takada@oici.jp (R.T.); tasuku.nakabori@oici.jp (T.N.); uehara@oici.jp (H.U.); kazuyoshi.ohkawa@oici.jp (K.O.)

**Keywords:** pancreatic cancer, FOLFOX, chemotherapy

## Abstract

Salvage chemotherapy for patients with unresectable pancreatic cancer (UR-PC) who have been treated with gemcitabine and nab-paclitaxel (GnP), and 5-fluorouracil (5-FU)/l-leucovorin (LV) plus nanoliposomal irinotecan (nal-IRI), has not been fully established. We retrospectively reviewed data from 17 patients with UR-PC who initiated 5-FU/l-LV plus oxaliplatin (FOLFOX) as salvage chemotherapy at our hospital between June 2020 and August 2021, after treatment with GnP and 5-FU/LV plus nal-IRI. The primary endpoint was tumor response. The secondary endpoints were progression-free survival (PFS) and adverse events (AEs). The response and disease control rates were 5.9% (1/17) and 17.6% (3/17), respectively. The median PFS was 1.8 months (range: 0.4–5.2 months). Eight patients (47.1%) experienced grade 3 nonhematologic AEs, while none experienced grade 3 hematologic AEs. Two patients with controlled disease had homologous recombination deficiency (HRD)-associated gene mutations in cancer panel testing. The FOLFOX regimen benefit for UR-PC patients treated with GnP and 5-FU/LV plus nal-IRI may be limited to patients with HRD-associated gene mutations.

## 1. Background

Pancreatic cancer (PC) is the seventh leading cause of cancer mortality, and accounts for almost as many deaths as cases because of its poor prognosis [1]. At the time of diagnosis, approximately 50% of patients are diagnosed with metastatic disease [2]. For patients with unresectable pancreatic cancer (UR-PC), systemic chemotherapy has been applied as the main treatment, resulting in the palliation of several cancer-related symptoms and the prolongation of survival time [2].

For patients with good performance status, the FOLFIRINOX regimen and the gemcitabine plus nab-paclitaxel (GnP) regimen, have been recommended as first-line treatments [3]. However, no clinical trials that have directly compared the two regimens and concluded which regimen is better have been published; the decision of which regimen to use is based on the patient’s age and general conditions in a clinical situation. Retrospective analyses have indicated that the benefit of the FOLFIRINOX regimen is limited to younger and fitter patients due to toxicity and the higher risk of febrile neutropenia [4]. Consequently, the GnP regimen is applied in more patients than the FOLFIRINOX regimen in clinical practice.

For patients with good performance status who received gemcitabine-based therapy as a first-line regimen, the 5-FU/l-leucovorin (LV) plus nanoliposomal irinotecan (nal-IRI) regimen was recommended as a second-line chemotherapy [3]. In the NAPOLI-1 trial, the 5-FU/LV plus nal-IRI regimen revealed a median progression-free survival (PFS) from 1.5 to 3.1 months (HR, 0.56; 95% CI, 0.41–0.75; *p* < 0.001) for patients who received the 5-FU/LV plus nal-IRI regimen, compared with patients who received 5-FU/LV monotherapy [5]. Updated analyses showed that the median OS for patients who received the 5-FU/LV plus nal-IRI regimen was also prolonged from 4.2 to 6.2 months (HR, 0.63; 95% CI: 0.47–0.85; *p* = 0.002); PFS was also prolonged [6]. After second-line treatments, the next steps are palliative and best supportive care or clinical trials, and no specific regimen for tertiary treatment is given in the guidelines [3]. Thus, additional treatment options to improve prognoses are necessary.

According to the National Comprehensive Cancer Network (NCCN) Clinical Practice Guidelines, the 5-FU/LV plus oxaliplatin (FOLFOX) regimen is recommended as one of the subsequent regimens for patients who received gemcitabine-based therapy [3]. In the CONKO-003 trial of patients with inoperable advanced PC, the use of the FOLFOX regimen as a second-line treatment, after the failure of first-line therapy with GEM, prolonged the median overall survival from 7.90 to 9.09 months compared with best supportive care (HR 0.50, *p* = 0.031) [7].

However, in the PANCREOX trial, overall survival was significantly shorter in modified FOLFOX6 group (oxaliplatin in addition to 5FU/LV) than in the control group (5FU/LV) (median of 6.1 months vs. 9.9 months; *p* = 0.02) [8]. Thus, salvage platinum-based chemotherapy has not been fully established. Recently, homologous recombination deficiency (HRD), which is a deficiency in the main system of double-stranded DNA repair, has been the focus of chemotherapy for pancreatic cancer. Tumor cells with HRD exhibit genomic instability due to the inability to repair double-stranded DNA adequately, resulting in the generation of point mutations and tumorigenesis [9]. HRD-related gene mutations have been shown to serve as predictive markers due to their sensitivity to DNA-damage-repair-targeted therapy, including platinum [10]. Approximately 10–20 other HRD-related genes have been reported [10]. Although platinum-based regimens seem to be preferred for patients with HRD-associated mutations, the prevalence of HRD is only approximately 20–40% [10,11]. However, the guidelines indicate that treatment based on genetic mutations is limited [3]. As a treatment that is not based on a specific genetic mutation, the efficacy of platinum-based regimens for patients with UR-PC who were previously treated with both the GnP regimen and the 5-FU/LV plus nal-IRI regimen is still not fully understood.

In this study, we investigated the efficacy and safety of the FOLFOX regimen as salvage chemotherapy for patients with UR-PC who had been previously treated with the GnP regimen and 5-FU/LV plus nal-IRI regimen.

## 2. Patients and Methods

We retrospectively evaluated the clinical data of 17 patients with pathologically diagnosed unresectable pancreatic adenocarcinoma, who initiated a platinum-based regimen as salvage chemotherapy between June 2020 and August 2021 after undergoing the GnP regimen and the 5-FU/LV plus nal-IRI regimen at our hospital. In this study, the FOLFOX regimen consisted of oxaliplatin 85 mg/m^2^ for 2 h of infusion and LV 200 mg/m^2^ for 2 h of infusion, followed by 5-FU continuous infusion of 2400 mg/m^2^ over 46 h, once every 2 weeks. The initial dose was reduced based on the discretion of the physicians. For each patient, data were extracted from medical records. The following clinical parameters were obtained: age, sex, Eastern Cooperative Oncology Group (ECOG) PS, primary tumor location, metastatic sites, and laboratory data (levels of white blood cells (WBCs), hemoglobin (Hb), platelets (Plt), and carbohydrate antigen 19-9 (CA19-9)). Imaging findings before and during chemotherapy, the details of the platinum-based regimen (dosages and schedules of therapeutic agents, treatment response, and toxicities), post-treatments, progression-free survival (PFS), overall survival (OS), and results of the Comprehensive Genome Profile (CGP) were assessed. The CGP was covered by the Japanese National Health Insurance. In this study, the following genes were defined as HRD-related genes, according to previous reports: BRCA1, BRCA2, PALB2, ATM, BAP1, BARD1, BLM, BRIP1, CHEK2, FAM175A, FANCA, FANCC, NBN, RAD50, RAD51, RAD51C, and RTEL1 [10]. Tumor responses were assessed according to the Response Evaluation Criteria in Solid Tumors (RECIST) ver. 1.1. CT images were captured every 8 ± 2 weeks or when clinical disease progression was suspected. Disease progression included an assessment of CT images and clinical disease progression without CT images. PFS was calculated from the date of FOLFOX initiation to the date of disease progression. OS was defined as the time from the date of FOLFOX initiation to the date of death. Survival curves of PFS and OS were constructed and evaluated using JMP Ver. 14.0 (SAS Institute, Cary, NC, USA). Adverse events were collected according to the Common Terminology Criteria for Adverse Events (CTCAE) ver. 5.0. Medical data were censored at the end of February 2022.

## 3. Results

### 3.1. Patient Characteristics

The patients’ characteristics are summarized in Table 1. The median age was 65 years (range: 39–76 years), and 52.9% of the patients were male. At the initiation of the first-line treatment (GnP), 52.9% of the patients were diagnosed with metastatic unresectable pancreatic cancer (UR-M). More than half of the patients had an ECOG score of 1 (52.9%). The median duration of pre-fluoropyrimidine-based chemotherapy was 3.4 months (range: 1.0–11.1 months). The initial dose of the FOLFOX regimen was reduced in thirteen patients. Thirteen patients were treated with FOLFOX as third-line chemotherapy, and four patients were treated with fourth-line chemotherapy. The median levels of WBCs, Hb, Plt, and CA19-9 at the initiation of the FOLFOX regimen are shown in Table 1.

### 3.2. Comprehensive Genome Profile (CGP)

Nine patients underwent a CGP, while the remaining eight patients did not due to insufficient histological samples, poor general conditions, or refusal to perform CGP. Three of the nine patients (33.3%) had pathogenic HRD-related gene mutations (BRCA1: 2 patients and RAD51C: 1 patient).

### 3.3. Tumor Response and Survival

**Figure 1:**
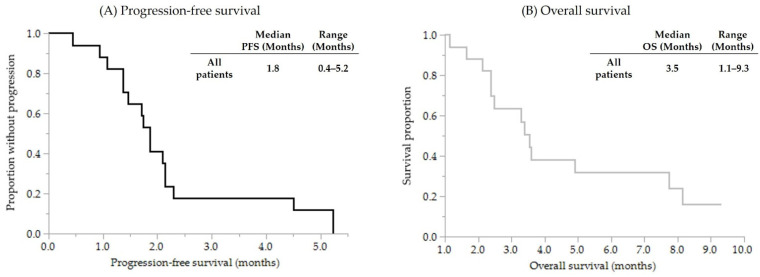
Kaplan–Meier survival curves for (**A**) progression-free survival and (**B**) overall survival.

One patient was classified as having a partial response, two patients as having stable disease, and thirteen patients as having progressive disease. One patient was not evaluated due to rapid clinical progression. According to these results, the response rate (RR) was 5.9% (1/17), and the disease control rate (DCR) was 17.6% (3/17). The median PFS was 1.8 months (range: 0.4–5.2 months). The median OS was 3.5 months (range: 1.1–9.3 months). The survival curves for OS and PFS are shown in Figure 1. Among the three patients with controlled disease, two patients had HRD-related gene mutations (one patient had a BRCA1 mutation, and the other had a RAD51C mutation). The remaining patient with controlled disease did not undergo CGP. In contrast, all six patients without HRD-related mutations were classified as having progressive disease. The median duration of pre-fluoropyrimidine-based chemotherapy was similar in both groups (DCR (+), 4.3 months (range: 3.7–4.7 months) vs. DCR (−), 2.5 months (range: 1.0–11.1 months), *p* = 0.256, Mann–Whitney U test).

### 3.4. Adverse Events

Hematological adverse events (AEs) were observed in eleven patients. No patients had grade 3/4 hematological AEs. Grade 1–2 leukopenia and neutropenia were observed in two patients. All eleven patients had grade 1–2 anemia. Grade 3 or 4 nonhematologic adverse events, including cholangitis (17.6%), anorexia (11.8%), fatigue (11.8%) and peripheral neuropathy (5.9%), were observed in eight patients (47.1%). No serious complications leading to death were observed.

## 4. Discussion

This study evaluated the efficacy and safety of the FOLFOX regimen as salvage chemotherapy for patients with UR-PC, who were previously treated with the GnP regimen and 5-FU/LV plus nal-IRI regimen. The median PFS in the present study (1.8 months) was similar to that in a previous study on FOLFOX as a second-line regimen (1.7–2.9 months) [7,12,13]. In terms of the best response, we observed that the response rate was 5.9% (1/17), and the disease control rate was 17.6% (3/17). A previous study of patients who received oxaliplatin, 5-fluorouracil and folinic acid combination regimens as second-line chemotherapy reported a partial response rate of 0–13.2%, PFS of 1.7–3.1 months, and OS from the start of second-line chemotherapy of 4.3–6.1 months [8,12,13]. Considering that the patients in the present study underwent FOLFOX as a third- or fourth-line regimen, these results suggest that FOLFOX has limited efficacy in UR-PC patients who received chemotherapy with GnP and 5-FU/LV plus nal-IRI.

Next, we focused on the nine patients who underwent CGP. Among them, three patients (33.3%) had HRD-related mutations. Two of the three patients who had HRD-related mutations achieved disease control, while the other six without HRD-related mutations did not. Germline or somatic HRD-related mutations are associated with striking sensitivity to platinum chemotherapy [10]. Patients with PC and mutations related to DNA damage repair genes often underwent platinum-based chemotherapy, regardless of whether the mutation was germline or somatic [14]. Based on a previous report, patients with either pathogenic somatic or germline HRD mutations, including mutations in BRCA1, BRCA2, PALB2, ATM, and CHEK2, are recommended to undergo HRD-targeted therapy [10]. These results suggest that FOLFOX as a salvage treatment may be more effective in patients with UR-PC who have HRD-related gene mutations.

Despite the small patient cohort, these findings indicate that salvage-line FOLFOX was well tolerated, and no grade-4 toxicities were observed. Regarding hematological AEs, we revealed that anemia, but not leukopenia or thrombocytopenia, was common. Conceivably, elevated white blood cells and decreased hemoglobin were caused by inflammation due to the progression of PC. Regarding nonhematological AEs, more than half of the patients had grade 3 nonhematologic AEs. However, no patient discontinued chemotherapy due to AEs. This study showed limited efficacy of this regimen, even in this specialized patient population who were treated with salvage chemotherapy after the GnP regimen and the 5-FU/LV plus nal-IRI regimen; this suggests that best supportive care may be preferable in these patients and that salvage chemotherapy with FOLFOX may be applicable to selected populations who have HRD-related gene mutations. Because this study is a preliminary, single-center, real-world clinical study, further research on a larger scale is necessary.

In conclusion, the benefit of the FOLFOX regimen for UR-PC patients treated with GnP and 5-FU/LV plus nal-IRI may be limited to patients with HRD-associated gene mutations, because only the patients with HRD-related mutations achieved disease control among the patients who underwent CGP.

## Figures and Tables

**Table 1:** Patient characteristics in the present study.

Number of patients, *n*	17
Median age (range), y.o	65 (39–76)
Sex	
Male, *n* (%)	9 (52.9%)
Female, *n* (%)	8 (47.1%)
Staging at diagnosis	
UR-LA, *n* (%)	7 (41.1%)
UR-M, *n* (%)	10 (58.9%)
Postoperative recurrence, *n* (%)	4 (23.5%)
Performance status	
0, *n* (%)	8 (47.1%)
1, *n* (%)	9 (52.9%)
Initial dose reduction, *n* (%)	13 (76.5%)
Treatment line	
3rd line, *n* (%)	13 (76.5%)
4th line, *n* (%)	4 (23.5%)
Median WBC (range), /μL	6240 (1020–14,680)
Median Hb (range), g/dL	12.7 (9.3–15.9)
Median Platelet (range), 10^4^/μL	26.9 (6.2–48.0)
Median CA19-9 (range), mg/dL	626 (3–22,700)

## Data Availability

Some of the participants in this study did not agree for their data to be shared publicly, and thus, supporting data are not available.

## References

[1] Sung H., Ferlay J., Siegel R.L., Laversanne M., Soerjomataram I., Jemal A., Bray F. (2021). Global Cancer Statistics 2020: GLOBOCAN Estimates of Incidence and Mortality Worldwide for 36 Cancers in 185 Countries. CA Cancer J. Clin..

[2] Mizrahi J.D., Surana R., Valle J.W., Shroff R.T. (2020). Pancreatic cancer. Lancet.

[3] Tempero M.A., Malafa M.P., Al-Hawary M., Behrman S.W., Benson A.B., Cardin D.B., Chiorean E.G., Chung V., Czito B., Del Chiaro M. (2021). Pancreatic Adenocarcinoma, Version 2.2021, NCCN Clinical Practice Guidelines in Oncology. J. Natl. Compr. Canc. Netw..

[4] Chan K.K.W., Guo H., Cheng S., Beca J.M., Redmond-Misner R., Isaranuwatchai W., Qiao L., Earle C., Berry S.R., Biagi J.J. (2020). Real-world outcomes of FOLFIRINOX vs gemcitabine and nab-paclitaxel in advanced pancreatic cancer: A population-based propensity score-weighted analysis. Cancer Med..

[5] Wang-Gillam A., Li C.P., Bodoky G., Dean A., Shan Y.S., Jameson G., Macarulla T., Lee K.H., Cunningham D., Blanc J.F. (2016). Nanoliposomal irinotecan with fluorouracil and folinic acid in metastatic pancreatic cancer after previous gemcitabine-based therapy (NAPOLI-1): A global, randomised, open-label, phase 3 trial. Lancet.

[6] Wang-Gillam A., Hubner R.A., Siveke J.T., Von Hoff D.D., Belanger B., de Jong F.A., Mirakhur B., Chen L.T. (2019). NAPOLI-1 phase 3 study of liposomal irinotecan in metastatic pancreatic cancer: Final overall survival analysis and characteristics of long-term survivors. Eur. J. Cancer.

[7] Pelzer U., Schwaner I., Stieler J., Adler M., Seraphin J., Dörken B., Riess H., Oettle H. (2011). Best supportive care (BSC) versus oxaliplatin, folinic acid and 5-fluorouracil (OFF) plus BSC in patients for second-line advanced pancreatic cancer: A phase III-study from the German CONKO-study group. Eur. J. Cancer.

[8] Gill S., Ko Y.J., Cripps C., Beaudoin A., Dhesy-Thind S., Zulfiqar M., Zalewski P., Do T., Cano P., Lam W.Y.H. (2016). PANCREOX: A Randomized Phase III Study of Fluorouracil/Leucovorin with or without Oxaliplatin for Second-Line Advanced Pancreatic Cancer in Patients Who Have Received Gemcitabine-Based Chemotherapy. J. Clin. Oncol..

[9] Lord C.J., Ashworth A. (2016). BRCAness revisited. Nat. Rev. Cancer.

[10] Park W., Chen J., Chou J.F., Varghese A.M., Yu K.H., Wong W., Capanu M., Balachandran V., McIntyre C.A., El Dika I. (2020). Genomic Methods Identify Homologous Recombination Deficiency in Pancreas Adenocarcinoma and Optimize Treatment Selection. Clin. Cancer Res..

[11] Pishvaian M.J., Bender R.J., Halverson D., Rahib L., Hendifar A.E., Mikhail S., Chung V., Picozzi V.J., Sohal D., Blais E.M. (2018). Molecular Profiling of Patients with Pancreatic Cancer: Initial Results from the Know Your Tumor Initiative. Clin. Cancer Res..

[12] Oettle H., Riess H., Stieler J.M., Heil G., Schwaner I., Seraphin J., Görner M., Mölle M., Greten T.F., Lakner V. (2014). Second-line oxaliplatin, folinic acid, and fluorouracil versus folinic acid and fluorouracil alone for gemcitabine-refractory pancreatic cancer: Outcomes from the CONKO-003 trial. J. Clin. Oncol..

[13] Zaanan A., Trouilloud I., Markoutsaki T., Gauthier M., Dupont-Gossart A.C., Lecomte T., Aparicio T., Artru P., Thirot-Bidault A., Joubert F. (2014). FOLFOX as second-line chemotherapy in patients with pretreated metastatic pancreatic cancer from the FIRGEM study. BMC Cancer.

[14] Perkhofer L., Golan T., Cuyle P.J., Matysiak-Budnik T., Van Laethem J.L., Macarulla T., Cauchin E., Kleger A., Beutel A.K., Gout J. (2021). Targeting DNA Damage Repair Mechanisms in Pancreas Cancer. Cancers.

